# Case Report: *Coxiella burnetii* vertebral osteomyelitis in a pigeon breeder: mNGS diagnosis of chronic Q fever

**DOI:** 10.3389/fmed.2025.1636778

**Published:** 2025-09-01

**Authors:** Minghui Song, Yumei Guo, Jiahao Hao, Cuiying Zheng, Huifen Zuo, Jiaqing Ye, Chenfeng Zhang, Feilong Chen, Zhongjun Feng, Hong Zhang, Zhenjun Zhao, Weili Gao, Lijie Zhang

**Affiliations:** ^1^Hebei Medical University Third Hospital, Shijiazhuang, China; ^2^Hebei Key Laboratory of Intractable Pathogens, Shijiazhuang Center for Disease Control and Prevention, Shijiazhuang, China; ^3^Hebei Yiling Hospital, Shijiazhuan, Shijiazhuang, China; ^4^Jiaxing Hospital of Traditional Chinese Medicine Affliated to Zhejiang Chinese Medical University, Jiaxing, China

**Keywords:** *Coxiella burnetii*, zoonosis, spinal infection, metagenomic next-generation sequencing, chronic Q fever

## Abstract

**Background:**

Isolated vertebral osteomyelitis represents an uncommon manifestation of chronic Q fever, posing significant diagnostic challenges. We report a case of *Coxiella burnetii*-induced spondylodiscitis confirmed via metagenomic next-generation sequencing (mNGS).

**Case report:**

A 52-year-old male with occupational avian exposure (pigeon breeder) presented with chronic low back pain persisting for over 1 year, refractory to serial epidural corticosteroid injections. Lumbar MRI demonstrated multifocal osteomyelitis (L3–L5) with associated intraspinal abscess. mNGS analysis of aspirate identified *C. burnetii*. Targeted dual antimicrobial therapy (vancomycin/doxycycline) induced progressive clinical resolution.

**Conclusion:**

*Coxiella burnetii*, the etiological agent of Q fever, exhibits global distribution and poses significant diagnostic challenges. Its clinical manifestations are frequently nonspecific, typically afebrile, and diagnosis is commonly delayed by months to years post-symptom onset. mNGS offers critical diagnostic utility for early identification and therapeutic intervention in rare spinal infections, thereby mitigating complication risks.

## Background

Q fever, a globally significant zoonosis, is caused by the obligate intracellular Gram-negative bacterium *Coxiella burnetii* (1). This pathogen exhibits high environmental stability and infectivity, with an extremely low infectious dose (1–10 organisms) sufficient to cause human infection. Q fever has a worldwide distribution but shows marked regional variations in clinical manifestations and epidemiological patterns (2). While domestic livestock constitute primary reservoirs for *C. burnetii*, humans represent accidental dead-end hosts that manifest clinical disease upon exposure. Acute Q fever predominates clinically, whereas chronic forms—which may emerge months to years post-exposure—are characterized by rare complications such as vertebral osteomyelitis, particularly in cases lacking predisposing vascular pathology or iatrogenic exposure (3). This report presents the case of *C. burnetii* vertebral osteomyelitis directly linked to chronic occupational exposure in pigeon breeding, establishing avian contact as a previously unrecognized risk factor for this zoonotic complication.

## Case presentation

A 52-year-old male patient presented with a 12-month history of progressive chronic low back pain without systemic symptoms such as fever or weight loss. Despite receiving repeated epidural corticosteroid injections and empirical anti-tubercular therapy, his symptoms progressively worsened. Five months preceding admission, the patient developed a cough and burning sensation in the left lower limb, accompanied by numbness on the lateral aspect of the right thigh. Lumbar magnetic resonance imaging revealed infectious lesions in L4 and L5 vertebrae. He was initially treated with a modified anti-tubercular regimen consisting of rifampicin, isoniazid, and lincomycin. After 3 months of treatment failure, the regimen was escalated to include streptomycin (750 mg intramuscularly daily) and levofloxacin (500 mg orally daily). Sixteen months after the initial onset of low back pain, the patient experienced a significant exacerbation of lumbosacral pain accompanied by cough and sputum production, prompting referral to our institution for further evaluation.

**Figure 1:**
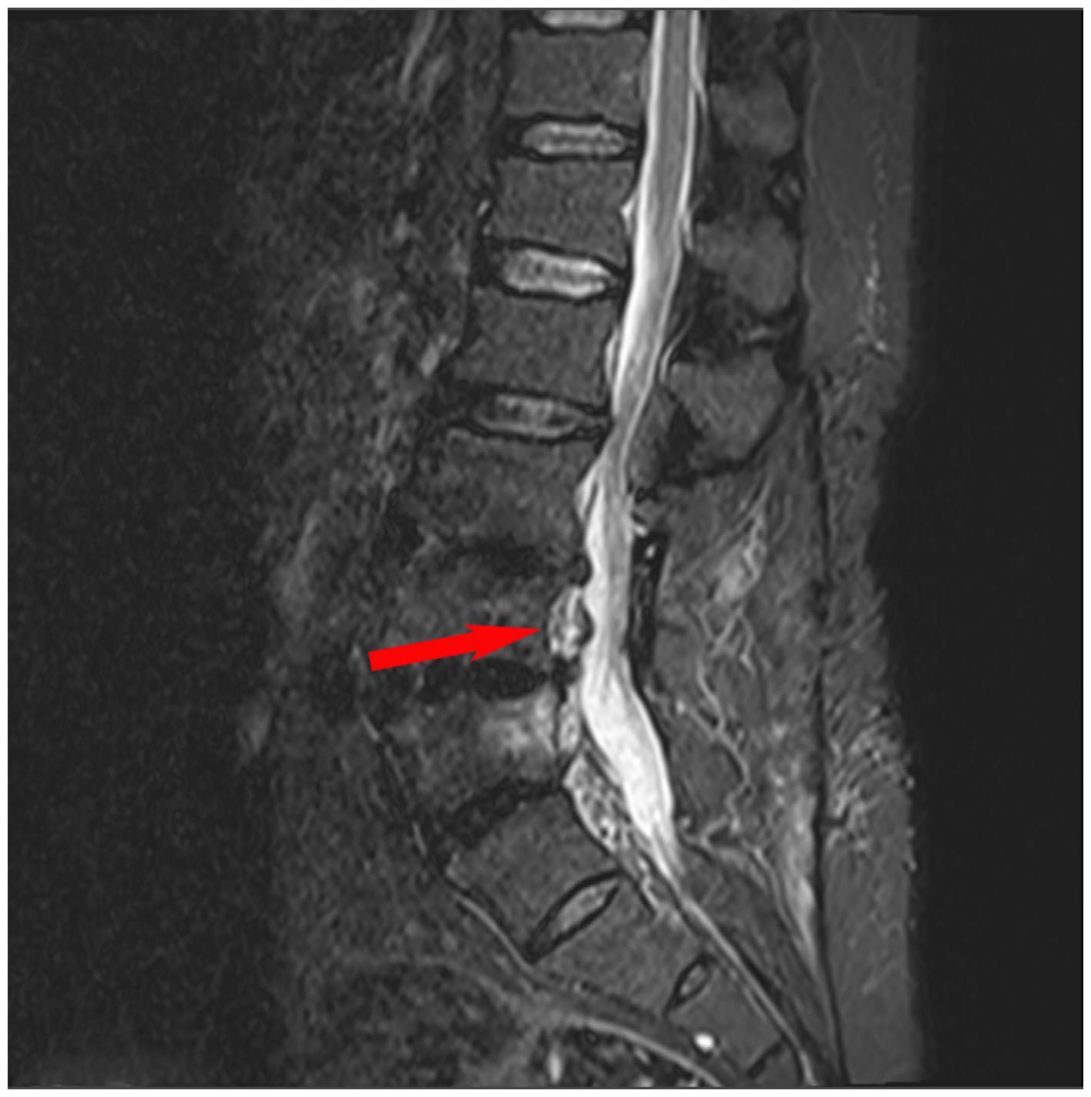
Sagittal magnetic resonance imaging (MRI) showing infection in the L3–L5 vertebrae with an intraspinal abscess of the patient before treatment.

**Figure 2:**
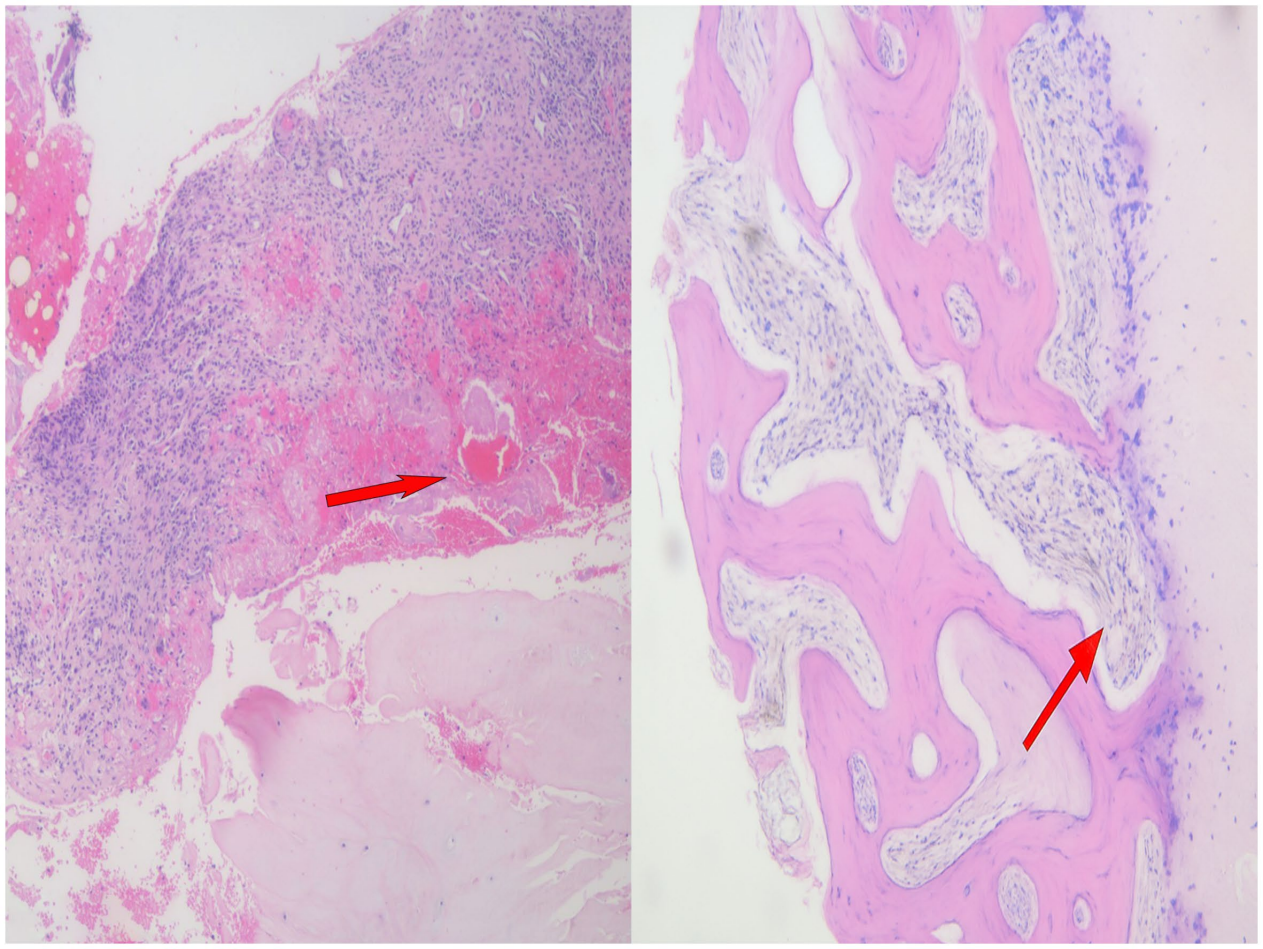
HE photomicrograph view (×100) showing chronic inflammation with degeneration and necrosis in the L5 vertebra before drug treatment.

On admission, the patient’s vital signs were stable: temperature 36.8 °C, pulse 78 beats/min, respiratory rate 18 breaths/min, and blood pressure 125/80 mmHg. Laboratory tests showed: Complete blood count revealed red blood cells at 5.01 × 10^12^/L, white blood cells at 5.77 × 10^9^/L, and platelets at 385 × 10^9^/L. Liver function tests showed an alanine aminotransferase (ALT) level of 34 U/L and an aspartate aminotransferase (AST) level of 23 U/L. Renal function tests revealed a creatinine level of 70 μmol/L and a blood urea nitrogen (BUN) level of 5.2 mmol/L, both within normal limits. Inflammatory markers: erythrocyte sedimentation rate (ESR) 16 mm/h, C-reactive protein (CRP) 11.77 mg/L. Lumbar spine Magnetic resonance imaging (MRI) revealed L3–L5 vertebral osteomyelitis with an intraspinal abscess (Figure 1). Comprehensive systemic evaluations yielded no abnormalities. Initial microbiological investigations, including serial blood cultures, acid-fast bacilli (AFB) staining were negative for pathogenic organisms. Histopathological analysis of lumbar tissue exhibited chronic inflammatory infiltrates with degenerative changes and necrotic foci at L5 (Figure 2). Upon admission, the patient received empirical antimicrobial therapy with piperacillin—tazobactam combined with levofloxacin while awaiting definitive microbiological results. To further identify the causative pathogen, metagenomic next-generation sequencing (mNGS) was performed on the vertebral aspirate using the BioelectronSeq 4000 platform. The mNGS analysis detected *Coxiella burnetii* with 1,125 sequence reads and 3.64% genome coverage. The diagnosis of *C. burnetii*-induced vertebral osteomyelitis was established based on the convergence of microbiological, histopathological, clinical, and radiological findings.

**Figure 3:**
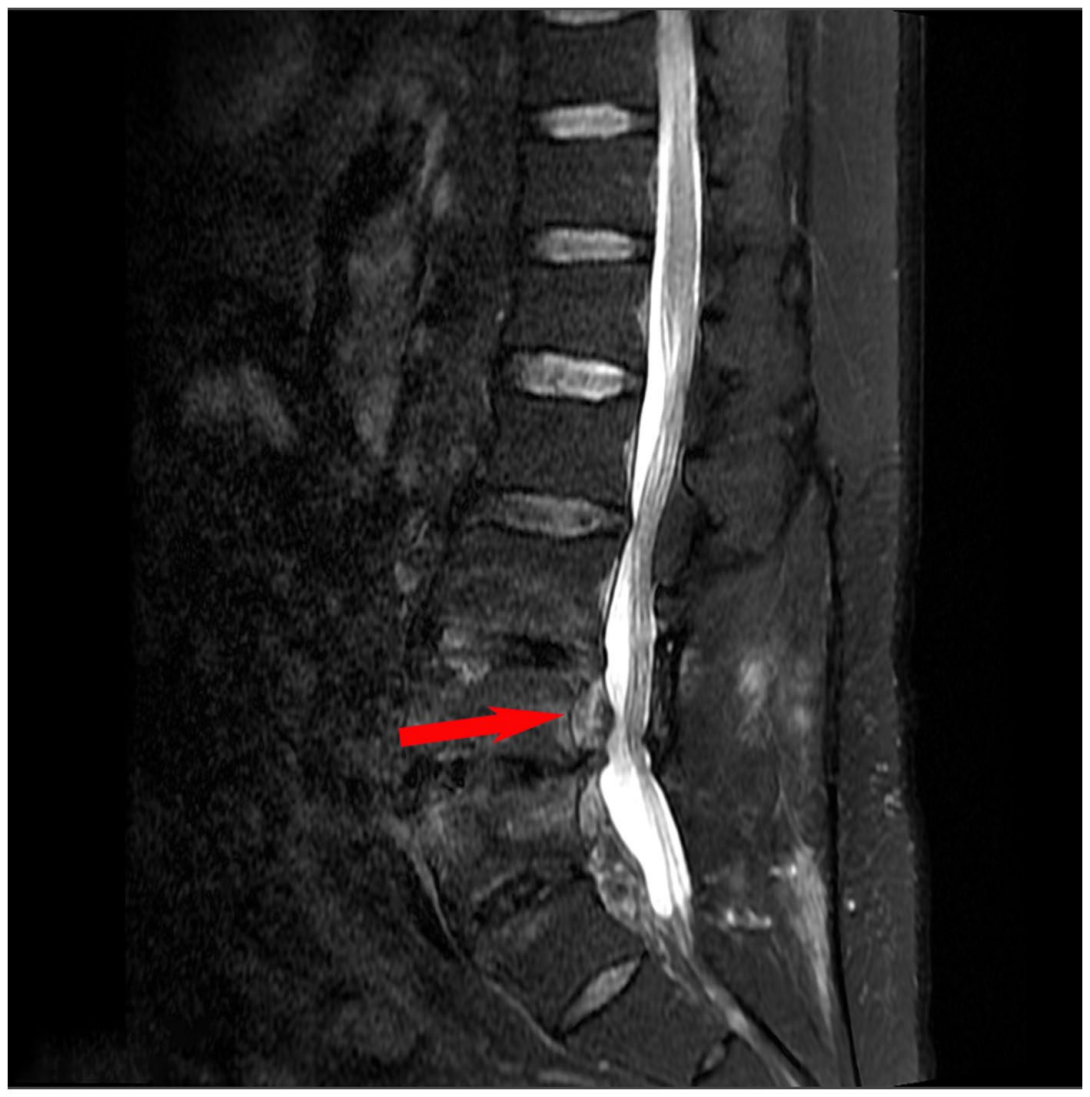
Sagittal magnetic resonance imaging (MRI) showing infection in the L3–L5 vertebrae with an intraspinal abscess of the patient after treatment.

**Figure 4:**
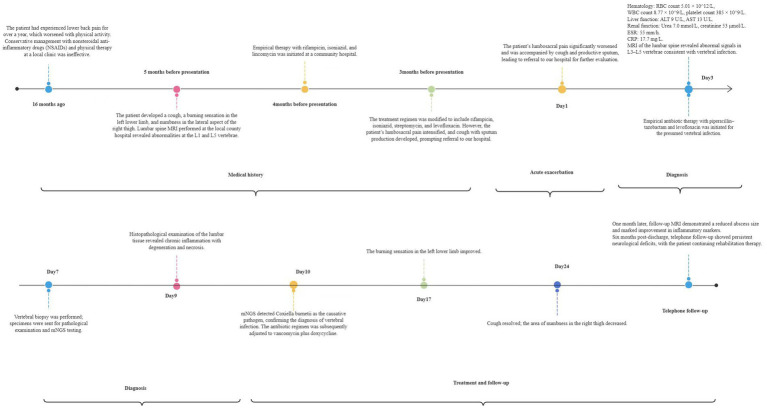
Chronological timeline summarizing the patient’s clinical course.

Following confirmation of *C. burnetii* infection by mNGS, the antimicrobial regimen was transitioned to vancomycin plus doxycycline. Adjunctive therapies included: oxycodone-acetaminophen (5 mg/6 h) analgesia, thromboprophylaxis, Xihuang capsules and estazolam (1 mg/nocte) for insomnia. By day 7 after adjustment of the therapeutic regimen, the burning sensation in the left lower extremity had diminished; by day 14, the cough had resolved and the area of numbness over the right lateral thigh had decreased. After 4 weeks of treatment, inflammatory markers improved markedly, with the erythrocyte sedimentation rate falling to 14 mm/h. Follow-up lumbar MRI (Figure 3) demonstrated a reduction in the volume of the epidural abscess and alleviation of low back pain. At discharge, vancomycin was discontinued, and doxycycline was continued for 17 months. At the six-month telephone follow-up, despite relief of back pain, the patient exhibited significant spinal nerve impairment due to delayed treatment: he required handrail support when climbing stairs, was unable to stand on tiptoe to retrieve objects, and had lost the ability to bear heavy loads. He is currently undergoing rehabilitation therapy. The clinical timeline is summarized in Figure 4.

## Discussion

Q fever is a zoonotic disease caused by *C. burnetii*, a Gram-negative, obligate intracellular bacterium. Domestic ruminants such as goats, sheep, and cattle are well-established reservoirs and primary sources of human infection (4). Transmission typically occurs through inhalation of pathogen-containing aerosols or direct contact with infected animals and their excreta. Occupational exposure constitutes a significant risk factor, particularly among livestock handlers, veterinarians, abattoir workers, and other professions involving close animal contact. Although pigeons are not conventional reservoirs of *C. burnetii*, the patient reported a history of long-term pigeon breeding in a humid, unhygienic environment. Avian species may acquire the pathogen through tick vectors or environmental contamination from infected mammals. This atypical exposure likely facilitated transmission through: (1) tick vectors infesting avian hosts, (2) direct contact with infected birds, or (3) inhalation of aerosolized excreta containing environmentally persistent *C. burnetii*. The diagnostic process in this case extended over a year, with initial misdiagnoses of degenerative spinal disorders and tuberculous spondylitis resulting in delayed treatment. Definitive diagnosis was ultimately achieved through mNGS of vertebral aspirate, which detected *C. burnetii* (1,125 sequence reads; 3.64% genome coverage). A literature review covering the period from 1990 to 2023 identified only 12 reported cases of isolated *C. burnetii* spinal infections. All documented cases highlight the diagnostic challenges posed by nonspecific symptomatology, with symptom duration ranging from months to years prior to diagnosis, frequently leading to misdiagnosis and therapeutic delays (5). Although Q fever classically affects individuals with livestock exposure, this case underscores the infection risk for residents near livestock-dense areas and companion animal handlers (e.g., pigeon breeders). Clinicians should consider *C. burnetii* infection in patients with chronic unexplained back pain or systemic symptoms, especially those with occupational or environmental exposure to zoonotic reservoirs. Targeted laboratory testing, including serological assays and advanced molecular diagnostics such as mNGS, should be pursued when epidemiological and clinical features suggest possible Q fever.

Approximately 1–5% of patients infected with *C. burnetii* develop chronic Q fever (6). Chronic Q fever vertebral osteomyelitis, with or without concomitant vascular infection, manifests as an indolent disease in which fever is not a predominant symptom. The most common clinical manifestation is nonspecific back pain, which is frequently misattributed to alternative etiologies, resulting in delayed diagnosis. Current diagnostic criteria for chronic Q fever primarily depend on serological assays and molecular detection of pathogen DNA in blood or tissue specimens (7), whereas successful bacterial isolation via conventional culture remains rare. Unfortunately, this case did not undergo Coxiella-specific serological testing nor genus-specific PCR on the vertebral aspirate, representing a significant diagnostic limitation and failing to meet the classical serology-based criteria for Q fever. In this context, metagenomic next-generation sequencing highlighted its strength in identifying rare pathogens. mNGS of the vertebral aspirate successfully detected *C. burnetii*, providing clinicians with definitive etiological evidence. As an emerging molecular diagnostic tool, mNGS offers clear advantages in the evaluation of infections of unknown origin. Unlike conventional culture-based methods, mNGS does not depend on pathogen growth and can, within a short timeframe, detect virtually all microorganisms present in clinical specimens—including viruses, bacteria, fungi, and parasites. Its high throughput and sensitivity make it particularly promising for complex infections, especially when traditional methods fail to identify the causative agent. In the present case, the successful identification of *C. burnetii* in vertebral puncture fluid via mNGS provided clinicians with definitive etiological confirmation.

The management of Q fever generally necessitates an extended regimen of combination antimicrobial therapy, spanning 18–24 months, with contemporary guidelines strongly advocating the use of hydroxychloroquine and doxycycline (8). For chronic Q fever, particularly when focal infections are present, guideline-recommended therapy includes at least two antibiotics, such as doxycycline plus hydroxychloroquine. However, because this patient had received prolonged rifampin therapy (approximately 3 months)—a potent cytochrome P450 enzymes (CYP3A4/2C8) inducer known to significantly reduce hydroxychloroquine plasma concentrations and markedly increase the risk of treatment failure (9)—the hydroxychloroquine-doxycycline regimen was not used. Instead, vancomycin (to cover potential pyogenic bacteria) was combined with doxycycline (the primary anti-*C. burnetii* agent). Vancomycin is ineffective against the intracellular *C. burnetii* and serves mainly to prevent or treat possible concomitant bacterial infections.

This case underscores the critical need to prioritize infectious etiologies in the differential diagnosis of patients presenting with persistent low back pain and concomitant neurological deficits, necessitating prompt imaging and advanced pathogen detection technology. Particularly in culture-negative spondylitis with a history of animal exposure, *C. burnetii* infection should be considered. While isolated spinal infections typically exhibit favorable prognoses, those caused by *C. burnetii*—particularly when complicated by vascular involvement—are associated with significant mortality, with an estimated 25% fatality rate attributed to Q fever-related vascular complications (10). Therefore, timely and accurate pathogen identification is clinically imperative, particularly for fastidious organisms such as *C. burnetii*. mNGS enables detection of low-abundance or intractable pathogens, thereby serving as a pivotal tool for early etiological clarification and targeted intervention in spinal infections of undetermined origin.

We present a rare case of isolated *C. burnetii* vertebral osteomyelitis in an atypical host (pigeon breeder), definitively diagnosed via mNGS after prolonged diagnostic delay. This case underscores the importance of considering Q fever in patients with culture-negative spondylitis and a history of animal exposure—even when the animals involved are not typical *C. burnetii* reservoirs. Furthermore, it highlights the unique value of mNGS for the early diagnosis and management of deep-seated infections caused by rare, fastidious, or atypical pathogens such as *C. burnetii*, providing a valuable reference for similar challenging cases.

## Data Availability

The original contributions presented in the study are included in the article/supplementary material, further inquiries can be directed to the corresponding authors.
